# Assessment of knowledge and practices regarding taeniasis and cysticercosis in Pak Chong, Nakhon Ratchasima, Thailand: A cross-sectional study

**DOI:** 10.1371/journal.pone.0307240

**Published:** 2024-07-15

**Authors:** Wuttipong Phumrattanaprapin, Nitima Tatiya-apiradee, Pattana Jantaban, Wiriya Mahikul

**Affiliations:** 1 Princess Srisavangavadhana College of Medicine, Chulabhorn Royal Academy, Bangkok, Thailand; 2 Program in Veterinary Technology, Faculty of Technology, Udon Thani Rajabhat University, Udon Thani, Thailand; 3 U-VET and Exotic Animal Hospital Rangsit, Bangkok, Thailand; Zambia Ministry of Health, ZAMBIA

## Abstract

**Background:**

Taeniasis and cysticercosis are parasitic infections caused by *Taenia* spp., mainly transmitted through the consumption of undercooked pork. Prevention requires increasing knowledge and awareness, improving meat inspection and hygiene, and promoting safe food handling and sanitation. The aim of this study was to evaluate the knowledge and practice (KP) of residents in Pak Chong District, Nakhon Ratchasima, Thailand, regarding taeniasis and cysticercosis.

**Methods:**

A cross-sectional study was conducted in Pak Chong District, Nakhon Ratchasima, Thailand, and its 11 subdistrict municipalities. Study participants were selected using a stratified random sampling design. A validated questionnaire (Cronbach’s alpha = 0.70) was used to collect socio-demographic information and assess knowledge and practices related to taeniasis and cysticercosis. Descriptive statistics and multiple linear regression were used for the analysis.

**Results:**

Of the 360 survey respondents, 65.0% (n = 234) were women, 82.2% (n = 296) were aged under 60 years, 99.2% (n = 357) identified as Buddhist, 87.5% (n = 315) had less than a bachelor’s degree education level, 54.2% (n = 195) had monthly family income ≥10,000 Thai baht, 10.6% (n = 38) were unemployed, and 26.1% (n = 93) lived in a town municipality. The survey revealed that 98.3% (n = 354) of participants were categorized as having less accurate knowledge and 83.6% (n = 301) were classified as more frequently engaging in correct prevention practices. Our study revealed that pig farmers (1.7%) occasionally permitted the pigs to forage freely. The results of multiple linear regression analysis revealed that knowledge scores were positively associated with household income ≥10,000 Thai baht (*β_adj_* = 1.50, 95% confidence interval [CI] 0.65 to 2.36). Practice scores were negatively associated with age over 60 years (*β_adj_* = −1.77, 95% CI −3.14 to −0.40) and living in a subdistrict municipality (*β_adj_* = −2.58, 95% CI −3.77 to −1.39). There was no association between KP regarding taeniasis and cysticercosis in the population of Pak Chong.

**Conclusions:**

Overall, participants’ knowledge was lacking. Public education interventions are recommended to improve knowledge among residents with low socioeconomic status. These findings can inform the development of targeted interventions and educational programs in Pak Chong District, especially among elderly people in subdistrict municipalities, to improve practices for the prevention and control of these parasitic infections.

## Introduction

Taeniasis is a parasitic infection caused by ingestion of undercooked pork contaminated with the tapeworm *Taenia solium*. Taeniasis poses a serious risk, leading to cysticercosis in individuals carrying the tapeworm and their close contacts [1]. Cysticercosis occurs when humans accidentally ingest *Taenia* eggs, causing tissue infection with the larval stage, *Cysticercus cellulosae*. *T*. *solium* cysticercosis is one of the most lethal parasitic diseases and the most important foodborne parasite, causing substantial health and economic burdens globally [2]. Both humans and pigs acquire cysticercosis through ingestion of *T*. *solium* eggs via the fecal-oral route [3]. Upon ingestion, the covering of the eggs is digested in the stomach, hatching the larval form (*C*. *cellulosae*). These larvae have the ability to penetrate the mucosal lining and enter both blood vessels and the lymphatic system and to disseminate throughout various tissues in the body, with a particular preference for the brain, muscle, skin, liver, lungs, and heart [4]. Pigs become infected when they are raised in unsanitary environments where they can access human feces [5].

These parasitic infections are serious public health concerns in endemic areas, especially in many developing countries, and have a detrimental effect on the well-being of millions of people worldwide. The World Health Organization recognizes taeniasis as one of the neglected tropical diseases that disproportionately affect impoverished populations worldwide [6, 7]. Endemic to various developing countries where pigs are raised as a food source, taeniasis and cysticercosis caused by *T*. *solium* are prevalent [8, 9]. In remote border communities where there is a prevalent lack of proper hygiene standards, people often engage in the common practice of consuming raw or partially raw dishes or vegetables and unwashed fruits, thereby exposing themselves to the risk of foodborne parasitic infections [10]. In Thailand, particularly along the Thailand–Myanmar border, taeniasis continues to be a common medical concern [11]. Recently, studies were conducted between 2011 and 2013 in three villages of Tha Song Yang District in Tak Province, located in northern Thailand. This province is situated along the Moei River, serving as a border between Thailand and Myanmar. During the study, a total of 983 fecal samples were examined using the Kato thick smear technique. The results revealed a prevalence of *Taenia* species eggs, with approximately 2.8% (28 out of 983) of the samples testing positive for these eggs [7]. In 2011, a study was conducted in three provinces of Thailand, namely, Nan in the north, and Ubon Ratchathani and Khon Kaen in the northeast, aiming to diagnose taeniasis and other helminthic infections through microscopic stool examinations. The study yielded notable results, indicating the presence of *Taenia* eggs in 2% of the samples from Nan, 3.7% from Ubon Ratchathani, and 0.9% from Khon Kaen [12]. In the present study area, Nakhon Ratchasima province in Northeast Thailand, a total of 199 fecal samples were tested for intestinal parasite eggs using the Mini Parasep SF fecal parasite concentrator during August to October 2015. Of all samples examined, 10 (5.03%) were positive for intestinal parasites including *Opisthorchis viverrini* (2.01%), followed by *Strongyloides stercoralis* (1.51%), hookworm (0.5%), and *Entamoeba coli* (0.5%); 1 of 199 samples (0.5%) were positive for *Taenia* spp. [13]. Intestinal helminthiases were screened using the Kato thick smear technique in the same area. A total of 209 stool samples were collected from people in Wang Sai subdistrict of Pak Chong district (May 2012) and Tanod subdistrict (July 2013) of Mueang Nakhon Ratchasima, Thailand. The results revealed that participants were infected with intestinal helminths, predominantly hookworm (4.31%, 9/209) followed by *S*. *stercoralis* (1.44%, 2/209) and *Taenia* spp. (0.48%, 1/209) [14]. Preventing the spread of these diseases within communities is challenging, emphasizing the importance of community-level control measures for cysticercosis [15, 16]. Although these diseases are preventable and treatable, control of taeniasis and cysticercosis requires a comprehensive understanding of their epidemiology, transmission, and prevention.

Human cases of taeniasis and cysticercosis are often linked to various factors, including consuming undercooked or raw pork infested with *T*. *solium* cysticerci; open defecation; having free-foraging pigs at home; use of sewage sludge as fertilizer on pastures; and use of open water sources such as rivers, streams, wells, and lakes without proper boiling or decontamination measures [7, 17, 18]. Additionally, the lack of knowledge about porcine and human cysticercosis [19–21], along with poor practices such as raw meat consumption, backyard slaughter, and poorly maintained latrines that allow pigs access to human feces, can lead to human ingestion of *T*. *solium* eggs [22, 23]. Moreover, free-roaming pigs, lack of meat inspection, lack of disease knowledge, and sewage spillage are associated with the epidemiology of taeniasis and neurocysticercosis [20].

In developing countries, several approaches have been attempted to control taeniasis/cysticercosis, emphasizing the crucial role of community awareness in disease control and eventual eradication. These include mass treatment for both humans and pigs to reduce taeniasis, the promotion of proper sanitation practices to minimize open-field defecation, meat inspection procedures at slaughterhouses, and health education initiatives [24]. The identification and treatment of individuals carrying the *Taenia* parasite play a crucial role in controlling the disease. This can be achieved through mass treatment programs or targeted health education campaigns that encourage individuals to self-report as *Taenia* carriers, followed by appropriate treatment [24]. Because the transmission of taeniasis and neurocysticercosis is influenced by various behavioral factors, health education plays an important role in reducing the spread of these conditions. Health education has been demonstrated to effectively reduce porcine cysticercosis and improve pig-rearing practices [25, 26].

Only a few studies have determined the knowledge, attitudes, and practices (KAP) related to *T*. *solium* cysticercosis and taeniasis. One study conducted in Tanzania revealed that participants had a good level of knowledge about porcine cysticercosis, but the practices of most participants were found to be inappropriate [27]. Another study conducted in a south Indian community revealed a significant lack of knowledge regarding the spread of taeniasis and neurocysticercosis. Furthermore, there was a noticeable absence of appropriate hygiene and sanitation practices [28]. In Ethiopia, the predicted proportion of self-reported taeniasis was moderately high, indicating a substantially high level of knowledge and positive attitudes; however, practices were found to be inferior [22]. Comprehensive evaluation of such knowledge and practice (KP) in the context of Thailand in lacking.

The aim of this study was to evaluate the KP of residents in Pak Chong District, Nakhon Ratchasima, Thailand, regarding taeniasis and cysticercosis. By investigating the KP of the target population, we sought to gain insight into the current causes of these diseases. Additionally, we aimed to identify the risk factors, including demographic characteristics, associated with KP concerning taeniasis and cysticercosis in town and subdistrict municipality areas of Pak Chong District.

## Methods

### Setting

This study was conducted in Pak Chong, Nakhon Ratchasima, located in Northeast Thailand, comprising individuals of Thai nationality whose names were registered in the household registry (123,382 participants, 49.69% men) including Town municipality and Subdistrict municipalities. Pak Chong Subdistrict (Town municipality), situated in Pak Chong District, Nakhon Ratchasima Province, is the principal subdistrict, encompassing a vast area. Its populace predominantly engages in agricultural pursuits, cultivating various crops and engaging in livestock farming, particularly poultry, swine, and dairy cattle, with sizable hatcheries dotting primary commercial and industrial zones. These establishments predominantly cluster within the downtown vicinity, constituting a commercial hub. An efficient transportation network, comprising automobiles, railways, and highways, facilitates seamless connectivity. Pak Chong District is a major tourist destination in Thailand’s northeastern region. Other subdistricts (Subdistrict municipalities) within Pak Chong District lag behind Pak Chong District in terms of development and public utility accessibility. Some regions remain isolated, lacking access to electricity and clean water systems for domestic consumption. In the general community, 79,136 individuals are aged 18–60 years. The prevalence of infection with *Taenia* spp. is 0.48% (1/209) and 0.5% (1/199) [13, 14]. A total of 8064 individuals are pig farmers, with an overall pig population of 358,042, according to the Information Technology and Communication Centre.

### Data collection and participants

We conducted a cross-sectional survey in March 2023 among Thai people aged 18–60 years. We sought cooperation from the Pak Chong District Public Health Office in advance. Our request involved engaging village health volunteers (VHVs) to act as representatives for collecting questionnaire data from residents in the area. We obtained the collaboration of 30 VHVs, who were trained by research staff between March 20 and March 24, 2023 in the questionnaire collection process, review procedures, and establishing return protocols before approaching respondents. Once all 30 VHVs were competent in the questionnaire collection methods, they were dispatched to their respective areas of responsibility to randomly select participants, following the suggested criteria for questionnaire respondents. After ensuring the accuracy of participant selection, VHVs returned the questionnaires to the researchers the following day (between March 30 and March 31, 2023). Subsequently, we meticulously reviewed the questionnaires for accuracy and completeness. If any irregularities or errors were detected, we engaged with the responsible VHVs to correct or verify the data accordingly. The sample size was calculated using an infinite population proportion formula, n = Z^2^p(1-p)/d^2^, with estimated knowledge level 85% [27], 4% precision (d), and a 95% confidence level. Therefore, the minimum calculated sample size was 307. Considering a 25% non-response rate, the maximum calculated sample size was approximately 385. The response rate was calculated by dividing the number of people returning the questionnaire by the total number of people with access to the questionnaire, i.e., 360 out of 385 completed and returned the questionnaire, which is greater than the minimum calculated sample size, giving a 93.5% response rate. The sample sizes for each subdistrict, based on stratified random sampling using the proportion allocation method according to the population distribution, were as follows: one town municipality with rapid urbanization, including Pak Chong subdistrict: 26.1%; 11 subdistrict municipalities where poverty and poor sanitation are twice as prevalent in rural areas, including Klang Dong subdistrict: 7.2%, Chan Thuek subdistrict: 8.9%, Wang Katha subdistrict: 2.8%, Mu Si subdistrict: 6.7%, Nong Sarai subdistrict: 16.7%, Kha Nong Phra subdistrict: 7.2%, Pong Talong subdistrict: 2.5%, Khlong Muang subdistrict: 5.8%, Nong Nam Daeng subdistrict: 5.6%, Wang Sai subdistrict: 6.4%, and Phaya Yen subdistrict: 4.2%.

In each subdistrict, 385 individuals were randomly selected and a single questionnaire was administered individually. Residents of communities in Pak Chong District and pig farmers were included in the survey. Responses to individual questions were analyzed and a score was calculated for each respondent based on the questionnaire. Average scores were calculated for the general community. All participants in this study provided their written informed consent. A total of 360 survey participants met the inclusion criteria. The data was collected in a private condition and kept confidential.

### Measures

The questionnaire included informed consent and questions regarding sociodemographic characteristics and KP.

Sociodemographic variables included working age (18–30, 31–45, and 46–59 years) and older age (≥60 years), sex, marital status, income, education, occupation, province of residence, source of consumed water, pig farming system, source of pigs raised on the farm, source of pig feed, source of water, purpose of pig farming, and presence of a slaughterhouse.

KP was assessed regarding taeniasis and cysticercosis using 22 questions for knowledge and 13 questions for practices (the first question addressing practices was not included in the total score). Knowledge questions had possible responses of “Yes,” “No,” and “Don’t know.” Correct answers (Yes) were coded as 1 point whereas wrong answers (No/Don’t know) were coded as 0 points. The level of KP was categorized using modified Bloom’s cutoff of 80%, indicating more accurate knowledge and more frequent correct practices [29]. Total scores for knowledge were classified into two categories: “Less accurate” (scores of 0–17 points) and “More accurate” (scores of 18–22 points). Practices were measured using positive and negative questions. Positive questions were measured on a 5-point Likert scale, as follows: “Always” (5 points), “Very often” (4 points), “Sometimes” (3 points), “Rarely” (2 points), and “Never” (1 point). Negative questions were also measured on a 5-point Likert scale, as follows: “Always” (1 point), “Very often” (2 points), “Sometimes” (3 points), “Rarely” (4 points), and “Never” (5 points). Total scores for practices were classified into two categories: “Less frequent” (scores of 12–47 points) and “More frequent” (scores of 48–60 points).

To enhance the scientific rigor of this research, we adopted and modified several previous processes commonly used in questionnaire methods, according to previous studies [20, 28, 30], including translation and back translation, peer and expert review, and piloting. Initially, a questionnaire was developed and subsequently sent to three academic experts with expertise in parasitology, epidemiology, public health, and medical practices related to taeniasis and cysticercosis. To ensure the validity of each question, we used the index of item objective congruence with a cutoff value of ≥0.5. Following the completion of content validity assessment, we conducted a pilot test involving 30 individuals to ascertain reliability of the questionnaire, which was assessed using coefficient analysis. Specifically, Cronbach’s alpha coefficients were calculated for KP, resulting in values of 0.77 and 0.72, respectively.

### Statistical analysis

Data analysis was performed using Microsoft Excel (Microsoft Corporation, Redmond, WA, USA) for editing, sorting, and coding. A complete Excel file was then imported into IBM SPSS software version 27 for data analysis (IBM Corp., Armonk, NY, USA). The study population characteristics were described using descriptive statistics such as frequency, percentage, mean, and standard deviation. The general personal information of the sample group included variables such as sex, religion, education level, occupation, and source of consumed water. These variables were analyzed using frequency and percentage for categorical variables; continuous variables such as age, income, knowledge scores, and practice scores related to taeniasis and cysticercosis are presented as mean and standard deviation.

Univariate analyses, including independent samples *t*-tests and chi-squared tests, were conducted to explore the association between dependent and independent variables. In the present study, we used various statistical tests to compare differences among personal factors for variables with a normal distribution. Independent samples *t*-tests were used for variables with two groups, and one-way analysis of variance was used for variables with more than two groups. The statistical significance level used for hypothesis testing was set at 0.05. Pearson’s correlation analysis was applied to examine the relationship between respondents’ KP. Linear regression analysis was conducted to explore the factors that could affect practices related to taeniasis and cysticercosis. Only variables showing a statistically significant association with the dependent variable (p<0.05) in the univariate analyses were considered in the multiple linear regression model, which was conducted with 95% confidence intervals (CIs). The linearity assumption was evaluated by analyzing the residuals.

### Ethical statement

The studies involving human participants were reviewed and approved by the Human Research Ethics Committee Chulabhorn Research Institute. Formal ethics approval was granted on 27 January 2023 (Project Code: EC 001/2565), and it conformed to the ethics guidelines of the Declaration of Helsinki. Written informed consent was obtained from all participants and if the subjects are illiterate, informed consent was obtained from legal representative. This study is reported in compliance with the STROBE statement.

## Results

### Demographic characteristics

In total, 360 participants were enrolled in the questionnaire survey. Among interviewed participants, 26.1% were residents of the town municipality (Pak Chong) and 73.9% were from the 11 subdistrict municipalities (Table 1). The mean participant age was 46.95±13.31 years. The mean age of residents in the town municipality was not significantly different from the age of participants from subdistrict municipalities (p = 0.153), and most (65.0%) were women (60.6% for the town municipality and 66.5% for subdistrict municipalities, with no significant difference, p = 0.302). Among respondents, 99.2% were Buddhist (98.9% for town municipality and 99.2% for subdistrict municipalities, with no significant difference, p = 0.781). Respondents’ education levels ranged from no education (1.7%, 0.0% for town municipality and 2.3% for subdistrict municipalities) to bachelor’s degree (10.8%, 7.4% for town municipality and 12.0% for subdistrict municipalities) with no significant difference (p = 0.065). Most participants (54.2%) reported an average monthly household income ≥10,000 Thai baht (THB); the average monthly household income was much higher among residents in the town municipality (66.0%) than among those from subdistrict municipalities (50.0%) (p = 0.008)). Of the total, 78.3% respondents were government or non-government employees (75.5% for town municipality and 79.3% for subdistrict municipalities, without significance, p = 0.390), 90.5% drank bottled or containerized drinking water (75.5% for town municipality and 79.3% for subdistrict municipalities, without significance, p = 0.209), only 1.7% worked on a pig farm (0.0% for town municipality and 2.3% for subdistrict municipalities, without significance, p = 0.346), 98.3% had less accurate knowledge (96.8% for town municipality and 98.9% for subdistrict municipalities, without significance, p = 0.186), and 83.6% more frequently engaged in correct practices (92.6% for town municipality and 80.5% for subdistrict municipalities, with significance, p = 0.006) (Table 1).

**Table 1:** Demographic characteristics of participants (N = 360).

Variables	Town municipality n (%) (N = 94)	Subdistrict municipalities n (%) (N = 266)	Total n (%) (N = 360)	p-value
Sex				
Female	57 (60.6)	177 (66.5)	234 (65.0)	0.302
Male	37 (37.4)	89 (33.5)	126 (35.0)	
Age (years)				
18–30	9 (9.6)	45 (16.9)	54 (15.0)	0.153
31–45	28 (29.8)	66 (24.8)	94 (26.1)	
46–59	44 (46.8)	104 (39.1)	148 (41.1)	
≥60	13 (13.8)	51 (19.2)	64 (17.8)	
Religious affiliation				
Buddhist	93 (98.9)	264 (99.2)	357 (99.2)	0.781
Other	1 (1.1)	2 (0.8)	3 (0.8)	
Education level				
No education	0 (0.0)	6 (2.3)	6 (1.7)	0.065
Less than bachelor’s degree	87 (92.6)	228 (85.7)	315 (87.5)	
Bachelor’s degree	7 (7.4)	32 (12.0)	39 (10.8)	
Average household monthly income (THB)				
<10,000	32 (34.0)	133 (50.0)	165 (45.8)	0.008
≥10,000	62 (66.0)	133 (50.0)	195 (54.2)	
Occupation				
Unemployed	9 (9.6)	29 (10.9)	38 (10.6)	0.390
Farmer	14 (14.9)	26 (9.8)	40 (11.1)	
Government or non-government employee	71 (75.5)	211 (79.3)	282 (78.3)	
Drinking water source				
Bottled or containerized drinking water	91 (96.8)	251 (94.4)	342 (95.0)	0.209
Purified water	3 (3.2)	10 (3.8)	13 (3.6)	
Natural water	0 (0.0)	5 (1.9)	5 (1.4)	
Working on pig farm				
Yes	0 (0.0)	6 (2.3)	6 (1.7)	0.346
No	94 (100.0)	260 (97.7)	354 (98.3)	
Knowledge level				
More accurate	3 (3.2)	3 (1.1)	6 (1.7)	0.186
Less accurate	91 (96.8)	263 (98.9)	354 (98.3)	
Practice level				
More frequent	87 (92.6)	214 (80.5)	301 (83.6)	0.006
Less frequent	7 (7.4)	52 (19.5)	59 (16.4)	

### Characteristics of pig farming

**Fig 1:**
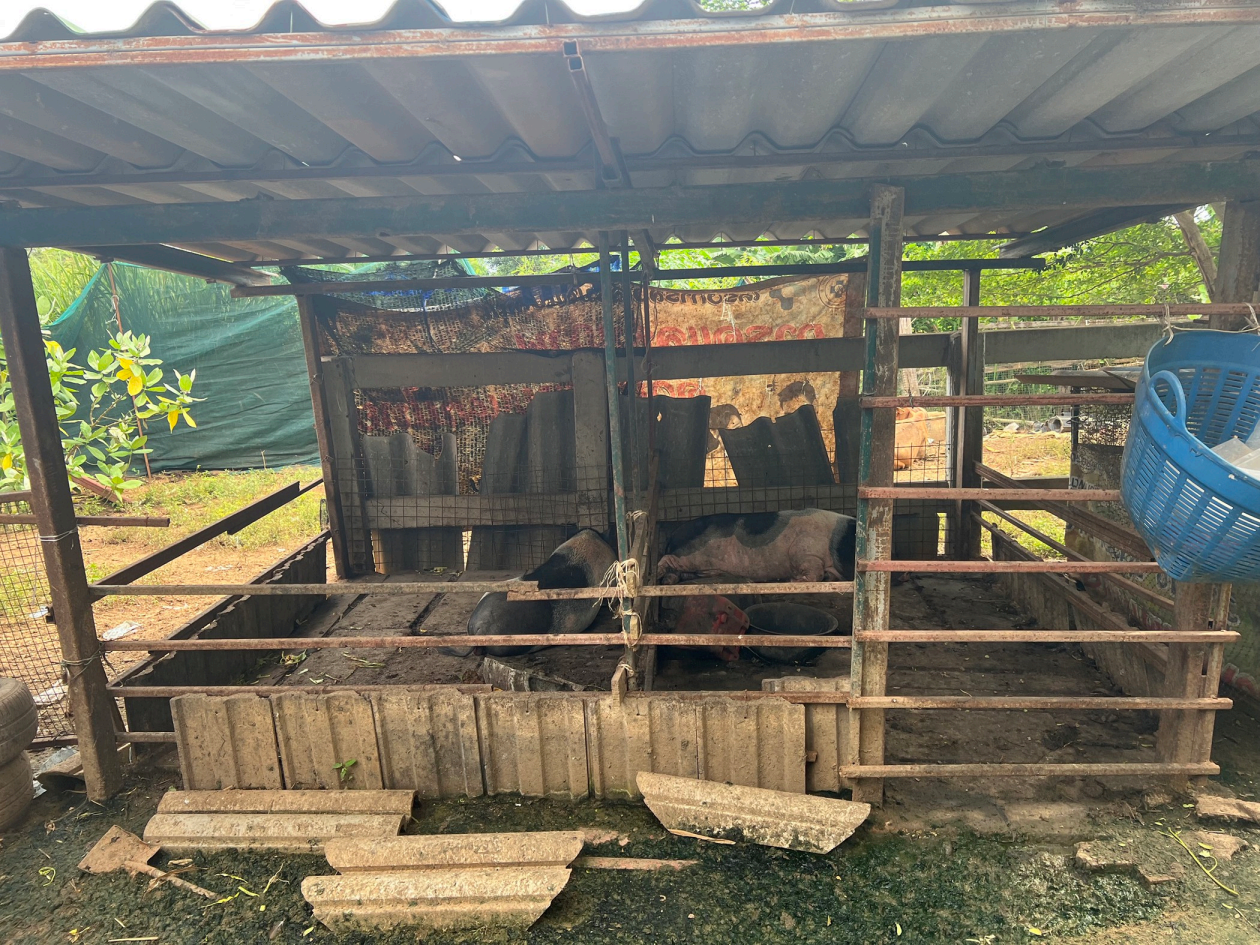
Pigpen construction.

Table 2 shows the characteristics of pig farming in Pak Chong, Nakhon Ratchasima, Thailand. The six included farmers collectively had a total of two to eight pigs. All of the pigs were raised in a closed-house system (Fig 1) and were sourced externally. Of the six farmers, five fed their pigs with food for human consumption/natural feed. Additionally, all pigs consumed tap water. Half of participants stated that their primary objective in pig farming was to sell the pigs, and none had a slaughterhouse on their own premises.

**Table 2:** Characteristics of pig farming in Pak Chong, Nakhon Ratchasima, Thailand.

Farmer	No. pigs	Pig farming system	Source of pigs raised on the farm	Food source	Water source	Purpose of pig farming	Slaughterhouse on farm
1	2	Closed	Outside	Human consumption/natural feed	Tap water	For sale	No
2	8	Closed	Outside	Human consumption/natural feed	Tap water	Consumption	No
3	6	Closed	Outside	Human consumption/natural feed	Tap water	For sale	No
4	2	Closed	Outside	Human consumption/natural feed	Tap water	Consumption	No
5	2	Closed	Outside	Human consumption/natural feed	Tap water	For sale	No
6	2	Closed	Outside	Delicatessen	Tap water	Other	No

### Knowledge

Regarding knowledge about taeniasis and cysticercosis (Table 3), participants from the town municipality more frequently correctly answered that taeniasis is a roundworm infection (53.2%) than participants from subdistrict municipalities (33.8%) (p <0.001). Participants residing in subdistrict municipalities provided more accurate responses (62.4%) regarding the relationship between ingesting raw pork containing encapsulated cysts and the development of mature pork tapeworms in the small intestine, compared with participants from the town municipality (48.9%) (p = 0.023). Participants from the town municipality demonstrated a higher level of accuracy (47.9%) in recognizing symptoms such as coughing, sneezing, and nasal discharge in patients with taeniasis, as compared with participants from subdistrict municipalities (34.2%) (p = 0.019). In terms of knowledge about prevention methods, 89.4% of individuals in the town municipality and 78.9% of those from subdistrict municipalities knew that taeniasis can be prevented by avoiding the consumption of raw pork (p = 0.025). Participants from the town municipality more often correctly answered that *T*. *solium* larvae can be found in both pigs and humans (80.9%) than those from subdistrict municipalities (68.0%) (p = 0.018). Few participants from the town municipality and subdistrict municipalities correctly answered that encysted larvae of *T*. *solium* can be found in the human small intestine (20.2% and 7.5%, p<0.001). Participants from the town municipality more correctly answered that taeniasis is treated solely by administering anthelmintic drugs (62.8%) compared with participants from subdistrict municipalities (31.2%, p<0.001).

**Table 3:** Questionnaire responses regarding knowledge about taeniasis and cysticercosis in Pak Chong, Nakhon Ratchasima, Thailand.

Statement	Residents in town municipality (N = 94), n (%)		Residents in subdistrict municipality (N = 266), n (%)		p-value
	Correct answer	Incorrect answer	Correct answer	Incorrect answer	
1. Taeniasis is a roundworm infection.	50 (53.2)	44 (46.8)	90 (33.8)	176 (66.2)	<0.001
2. *Taenia solium* (pork tapeworm) inhabits the small intestine of humans.	39 (41.5)	55 (58.5)	132 (49.6)	134 (50.4)	0.175
3. Consumption of raw or undercooked pork is the main cause of taeniasis.	83 (88.3)	11 (11.7)	233 (87.6)	33 (12.4)	0.858
4. Consuming cooked pork can still lead to taeniasis.	44 (46.8)	50 (53.2)	135 (50.8)	131 (49.2)	0.511
5. Encysted larvae of *T*. *solium* can be found in pork meat, appearing as small white cysts, containing encapsulated larvae.	63 (67.0)	31 (33.0)	175 (65.8)	91 (34.2)	0.828
6. Pork meat with encapsulated cysts can be safely consumed if cooked thoroughly before consumption.	28 (29.8)	66 (70.2)	81 (30.5)	185 (69.5)	0.904
7. Ingesting raw pork containing encapsulated cysts can lead to the presence of mature pork tapeworm in the small intestine.	46 (48.9)	48 (51.1)	166 (62.4)	100 (37.6)	0.023
8. Patients with taeniasis exhibit symptoms such as coughing, sneezing, and nasal discharge.	45 (47.9)	49 (52.1)	91 (34.2)	175 (65.8)	0.019
9. Taeniasis cannot be completely cured.	41 (43.6)	53 (56.4)	101 (38.0)	165 (62.0)	0.336
10. Free-range pig farming, (allowing pigs to forage naturally) increases the risk of pigs acquiring *T*. *solium* larvae.	58 (61.7)	36 (38.3)	156 (58.6)	110 (41.4)	0.604
11. Taeniasis can be prevented by avoiding the consumption of raw pork.	84 (89.4)	10 (10.6)	210 (78.9)	56 (21.1)	0.025
12. Taeniasis can be prevented by inspecting the quality of meat at slaughterhouses.	37 (39.4)	57 (60.6)	89 (33.5)	177 (66.5)	0.302
13. *T*. *solium* larvae can encyst in human muscles, lungs, heart, and even the brain.	38 (40.4)	56 (59.6)	100 (37.6)	166 (62.4)	0.627
14. *T*. *solium* larvae can be found in both pigs and humans.	76 (80.9)	18 (19.1)	181 (68.0)	85 (32.0)	0.018
15. Ingestion of raw pork is the pathway through which humans acquire larval cysts of *T*. *solium*.	7 (7.4)	87 (92.6)	22 (8.3)	244 (91.7)	0.801
16. Ingestion of food and water contaminated with *T*. *solium* eggs is another pathway through which humans acquire *T*. *solium* larval cysts.	56 (59.6)	38 (40.4)	160 (60.2)	106 (39.8)	0.922
17. Encysted larvae of *T*. *solium* can be found in the human small intestine.	19 (20.2)	75 (79.8)	20 (7.5)	246 (92.5)	<0.001
18. Patients harboring *T*. *solium* larvae in their bodies primarily exhibit gastrointestinal symptoms.	17 (18.1)	77 (81.9)	40 (15.0)	226 (85.0)	0.487
19. Patients with *T*. *solium* larvae in the brain may experience headaches and seizures, and this can be fatal.	40 (42.6)	54 (57.4)	102 (38.3)	164 (61.7)	0.473
20. Preventing taeniasis can be achieved by washing vegetables and fruits before consumption, as well as drinking clean water.	72 (76.6)	22 (23.4)	206 (77.4)	60 (22.6)	0.866
21. Taeniasis is treated solely by administering anthelmintic drugs.	59 (62.8)	35 (37.2)	83 (31.2)	183 (68.8)	<0.001
22. Taeniasis can cause epileptic seizures.	18 (19.1)	76 (80.9)	53 (19.9)	213 (80.1)	0.871

### Practices

Regarding practices related to taeniasis and cysticercosis (Table 4), participants residing in the town municipality exhibited a higher frequency of pork meat consumption (43.6%) than participants from subdistrict municipalities (27.8%) (p<0.001). Conversely, participants from subdistrict municipalities reported a higher frequency of annual anthelmintic medication intake (5.6%) than those from the town municipality (5.3%) (p = 0.007). Additionally, participants from the town municipality were more likely to wash vegetables and fruits with tap water (88.3% in both groups) than participants from subdistrict municipalities (67.3% and 68.0%, respectively; p<0.001). Participants from subdistrict municipalities tended to wash vegetables and fruits with water from natural sources (5.3% in both groups) more frequently than those from the town municipality (1.1% in both groups, p = 0.011 and 0.028, respectively). Furthermore, participants from the town municipality demonstrated a higher frequency of washing hands with clean water and soap after using the restroom and before handling food (79.8% and 72.3%) compared with participants from subdistrict municipalities (54.5% and 48.1%, respectively; p<0.001). Additionally, participants from subdistrict municipalities had a greater frequency of consuming water from natural sources, such as rivers (1.9%), compared with participants from the town municipality (1.1%) (p = 0.028).

**Table 4:** Frequency distribution of practices regarding taeniasis and cysticercosis in Pak Chong, Nakhon Ratchasima, Thailand.

Statement	Residents in town municipality (N = 94), n (%)	Residents in subdistrict municipalities (N = 266), n (%)	Total (N = 360), n (%)	p-value
1. Always consume pork meat	41 (43.6)	74 (27.8)	115 (31.9)	0.005
2. Always cook pork meat properly before consuming it	83 (88.3)	231 (86.8)	314 (87.2)	0.108
3. Always consume pork meat that contains larval cysts of *T*. *solium*	4 (4.3)	2 (0.8)	6 (1.7)	0.275
4. Always examine the appearance of pork meat before preparing it	67 (71.3)	161 (60.5)	228 (63.3)	0.083
5. Always take anthelmintic medication annually	5 (5.3)	15 (5.6)	20 (5.6)	0.007
6. Always defecate in open areas, such as bodies of water, soil, or pit latrines	2 (2.1)	3 (1.1)	5 (1.4)	0.063
7. Always wash vegetables with tap water	83 (88.3)	179 (67.3)	262 (72.8)	<0.001
8. Always wash fruits with tap water	83 (88.3)	181 (68.0)	264 (73.3)	<0.001
9. Always wash vegetables with water from natural sources	1 (1.1)	14 (5.3)	15 (4.2)	0.011
10. Always wash fruits with water from natural sources	1 (1.1)	14 (5.3)	15 (4.2)	0.028
11. Always wash hands with clean water and soap after using the restroom	75 (79.8)	145 (54.5)	220 (61.1)	<0.001
12. Always wash hands with clean water and soap before handling food	68 (72.3)	128 (48.1)	196 (54.4)	<0.001
13. Always drink water from natural sources, such as rivers	1 (1.1)	5 (1.9)	6 (1.7)	0.028

Table 5 presents the factors associated with knowledge (More accurate vs. Less accurate) and practices (More frequent vs. Less frequent), respectively. There were no factors associated with knowledge analyzed using the chi-square test. Among participants from the subdistrict municipality, 88.1% exhibited good practices less frequently and 71.1% exhibited good practices more frequently (p = 0.006). Among participants aged ≥60 years old, 30.5% had less frequent good practices, compared with 15.3% who had more frequent good practices (p = 0.037). However, there was no association between KP in each municipality.

**Table 5:** Factors associated with knowledge and practices among study participants regarding taeniasis and cysticercosis in Pak Chong, Nakhon Ratchasima, Thailand (N = 360).

Variable	Category	Knowledge			Practices		
		Less accurate n (%)	More accurate n (%)	p-value	Less frequent n (%)	More frequent n (%)	p-value
Area resident	Town municipality	91 (25.7%)	3 (50%)	0.186	7 (11.9%)	87 (28.9%)	0.006
	Subdistrict municipalities	263 (74.3%)	3 (50%)		52 (88.1%)	214 (71.1%)	
Sex	Female	232 (65.5%)	2 (33.3%)	0.189	40 (67.8%)	194 (64.5%)	0.622
	Male	122 (34.5%)	4 (66.7%)		19 (32.2%)	107 (35.5%)	
Age (years)	18–30	52 (14.7%)	2 (33.3%)	0.177	8 (13.6%)	46 (15.3%)	0.037
	31–45	94 (26.6%)	0 (0.0%)		15 (25.4%)	79 (26.2%)	
	46–59	146 (41.2%)	2 (33.3%)		18 (30.5%)	130 (43.2%)	
	≥60	62 (17.5%)	2 (33.3%)		18 (30.5%)	46 (15.3%)	
Religion	Buddhism	351 (99.2%)	6 (100.0%)	1.000	57 (96.6%)	300 (99.7%)	0.071
	Other	3 (0.8%)	0 (0.0%)		2 (3.4%)	1 (0.3%)	
Education level	Uneducated	5 (1.4%)	1 (16.7%)	0.051	2 (3.4%)	4 (1.3%)	0.523
	Lower (below bachelor’s degree)	312 (88.1%)	3 (50.0%)		51 (86.4%)	264 (87.7%)	
	Bachelor’s degree	37 (10.5%)	2 (33.3%)		6 (10.2%)	33 (11.0%)	
Average monthly household income (THB)	<10,000	161 (45.5%)	4 (66.7%)	0.419	26 (44.1%)	139 (46.2%)	0.766
	≥10,000	193 (54.5%)	2 (33.3%)		33 (55.9%)	162 (53.8%)	
Occupation	Unemployed	38 (10.7%)	0 (0.0%)	0.228	6 (10.2%)	32 (10.6%)	0.807
	Farmer	40 (11.3%)	0 (0.0%)		8 (13.6%)	32 (10.6%)	
	Government and non-government employee	276 (78.0%)	6 (100.0%)		45 (76.3%)	237 (78.7%)	
Working on pig farm	No	348 (98.3%)	6 (100.0%)	1.000	58 (98.3%)	296 (98.3%)	1.000
	Yes	6 (1.7%)	0 (0.0%)		1 (1.7%)	5 (1.7%)	
Knowledge level	Less accurate	-	-	-	59 (100.0%)	295 (98%)	0.595
	More accurate				0 (0.0%)	6 (2.0%)	

Pearson’s correlation analysis indicated no significant relationships (p = 0.587) with KP (r = −0.029). The results of univariate linear regression revealed that the mean knowledge score was lower among participants living in subdistrict municipalities (*β* = −0.98, 95% CI −0.98 to 0.02) and those with lower education levels (below bachelor’s degree) (*β* = −1.43, 95% CI −2.67 to −1.30), holding all other variables constant (Table 6). The mean knowledge score was higher among participants with household income ≥10,000 THB (*β* = 1.74, 95% CI 0.90 to 2.57). Good practice scores were lower in participants aged ≥60 years (*β* = −1.95, 95% CI −3.45 to −0.56) and those living in subdistrict municipalities (*β* = −2.68, 95% CI −3.88 to −1.48). Moreover, the results of multiple linear regression revealed that the mean knowledge score was higher in participants with household income ≥10,000 THB (*β_adj_* = 1.50, 95% CI 0.65 to 2.36), keeping all other variables constant (Table 6). Good practice scores were lower among participants aged ≥60 years (*β_adj_* = −1.77, 95% CI −3.14 to −0.40) and those living in subdistrict municipalities (*β_adj_* = −2.58, 95% CI −3.77 to −1.39). However, we found no association with KP regarding taeniasis and cysticercosis among the population of Pak Chong.

**Table 6:** Univariate and multiple linear regression models for the relationship between KP scores and independent variables for taeniasis and cysticercosis in Pak Chong, Nakhon Ratchasima, Thailand.

Variable	Category	Knowledge score						Practice score					
		*β*	95% CI	p-value	*β_adj_*	95% CI	p-value	*β*	95% CI	p-value	*β_adj_*	95% CI	p-value
Area resident	Town municipality	Ref			Ref			Ref			Ref		
	Subdistrict municipalities	−0.98	−1.94, 0.02	0.046	−0.82	−1.77, 1.42	0.095	−2.68	−3.88, −1.48	<0.001	−2.58	−3.77, 1.39	<0.001
Sex	Female	Ref						Ref					
	Male	0.05	−0.84, 0.94	0.916				−0.49	−1.62, 0.64	0.398			
Age (years)	<60	Ref						Ref			Ref		
	≥60	0.70	−0.41, 1.81	0.214				−1.95	−3.35, −0.56	0.006	−1.77	−3.14, −0.40	0.011
Religion	Buddhism	Ref						Ref					
	Other	0.88	−3.78, 5.54	0.711				−0.49	−10.59, 1.24	0.121			
Education level	Uneducated	Ref			Ref			Ref					
	Lower (below bachelor’s degree)	−1.43	−2.67, −1.30	0.031	−1.09	−2.37, 1.81	0.092	0.35	−1.28, 1.98	0.671			
	Bachelor’s degree	1.32	−0.03, 2.68	0.056				0.26	−1.48, 1.99	0.770			
Average monthly household income (THB)	<10,000	Ref			Ref			Ref					
	≥10,000	1.74	0.90, 2.57	<0.001	1.50	0.65, 2.36	<0.001	−0.18	−1.26, 0.90	0.743			
Occupation	Unemployed	Ref						Ref					
	Farmer	−0.39	−1.75, 0.95	0.563				−0.29	−2.01, 1.43	0.739			
	Government and non-government employee	0.36	−0.67, 1.39	0.492				0.67	−0.67, 1.98	0.313			
Working on pig farm	No	Ref						Ref					
	Yes	−2.84	−6.14, 0.46	0.091				1.21	−3.00, 5.43	0.572			
Knowledge level	Less accurate	-	-	-				Ref					
	More accurate				-	-	-	4.09	−0.10, 8.29	0.056			

CI, confidence interval.

## Discussion

Taeniasis is recognized as a serious public health issue in many Southeast Asian countries, including Cambodia, Indonesia, Japan, Laos, Malaysia, Nepal, Thailand, the Philippines, China, and Vietnam [31]. The disease carries a zoonotic risk as it can lead to cysticercosis in infected individuals. The primary mode of transmission for *T*. *solium* is through the consumption of raw or undercooked pork. In this study, we aimed to assess KP related to taeniasis and cysticercosis in the Nakhon Ratchasima province of Northeast Thailand, an area with an infection rate of *Taenia* spp. ranging from 0.48% to 0.5% [13, 14]. In Ubon Ratchathani province, also in Northeast Thailand, a higher prevalence of 3.7% was observed among 296 individuals [32]. Waikagul et al. (2006) reported a high prevalence of 5.9% among 1450 residents of 30 villages in the north of Thailand, as well as a prevalence of 2.8% among 1233 stool samples from 19 provinces in Northeast Thailand [33]. These findings support the higher incidence of taeniasis in northern and northeastern regions of the country, where the consumption of raw or undercooked meat is prevalent, contributing to the relatively high prevalence of this disease among residents of those areas [32]. In this study, 11.7% of participants reported consuming raw or undercooked meat with a frequency of sometimes to always.

This study employed a quantitative approach, specifically a survey on participants’ KP, to evaluate their level of KP regarding taeniasis and cysticercosis within a specific population at risk owing to their eating habits and cultural practices. The survey sample comprised a greater proportion of women (65%) than men (35%), similar to findings from a study by Ngowi et al. (2017) in three provinces of Burkina Faso where 53.8% of participants were women [25]. This sex distribution may be attributed to the involvement of women as both participants and village health volunteers (VHVs) during the study period, with women traditionally engaged in household activities while men worked outside the home. The mean age of participants was 46.95±13.31 years, but no significant correlations were found among sex, religion, and knowledge levels. Furthermore, most participants (87.5%) had lower education levels, primarily a primary education level, followed by a bachelor’s degree (10.8%) and no education (1.7%). This suggests that the participants in Pak Chong district, who predominantly reside in rural areas, face socioeconomic factors that necessitate terminating their education earlier so as to enter the workforce, as compared with residents of urban areas who have easier access to education. Notably, a greater level of knowledge was associated with a higher socioeconomic status, consistent with a study by Rahman et al. (2021) in Thailand and Laos, which found a significant association between participants’ knowledge of climate change and dengue and their education level and socioeconomic status [34]. Although not statistically significant, the general population below 60 years of age demonstrated higher levels of knowledge than those above 60 years of age. This finding underscores the need for well-designed health education initiatives targeting specific groups, particularly elderly people, focusing on topics such as hygiene, sanitation, and treatment.

A small percentage (1.7%) of participants randomly selected in this study were identified as pig farmers and used a smallholding pig production system for household consumption and selling pigs to slaughterhouses. These pig farmers collectively owned a total of two to eight pigs. Notably, five out of six pig farmers reported feeding their pigs with food for human consumption or natural feed such as vegetable scraps, banana peels, and morning glory. This practice poses a potential risk of contamination with *Taenia* eggs because these feed sources can be contaminated with feces containing the eggs of the parasite [35]. This finding aligns with those of a study by Kusolsuk et al. (2021) in Tak province, Thailand, which identified risk factors for infection with *Taenia* spp., including a history of raw or undercooked pork consumption, open defecation, and free-foraging pigs in the home. Our study revealed that pig farmers occasionally allowed their pigs to forage freely, which could increase the risk of pigs coming into contact with *Taenia* eggs, which can be present in surface water. Additionally, the use of sewage sludge as fertilizer on pastures has been identified as an important risk factor for cysticercosis [7]. This is consistent with the findings of Jansen et al. (2021), who conducted a systematic review on the survival and dispersal of *Taenia* eggs in the environment [36]. Similarly, Boone et al. (2007) reported that factors such as flooding of pastures, free access of cattle to surface water, and proximity to wastewater effluent, were associated with bovine cysticercosis in Belgian dairy and mixed herds [37]. A small proportion (1.4%) of our participants reported engaging in open defecation practices, such as in bodies of water, soil, or pit latrines, which could decrease the risk of egg dissemination into water and the environment.

In terms of knowledge, the survey results indicated that, on average, 98.3% of the population in Pak Chong district had lower knowledge levels regarding taeniasis and cysticercosis. This can be attributed to factors such as participants’ low level of education, cultural beliefs, and limited access to information. Similar findings were observed in a study conducted in Burkina Faso, where participants who had heard about cysticercosis were found to have better knowledge about the disease [38]. A lack of knowledge about the epidemiology of porcine and human cysticercosis contributes to behaviors that facilitate the transmission and maintenance of *T*. *solium* infections [19, 21]. However, we noted that the general population residing in the town municipality had a better understanding of taeniasis infection, including the morphology of *T*. *solium*, its symptoms, and the presence of *T*. *solium* larvae in both pigs and humans. These participants also had a greater awareness of the existence of cysts in patients compared with the general population residing in subdistrict municipalities. Conversely, individuals residing in subdistrict municipalities exhibited a better understanding of the causes of taeniasis, in comparison with those in town municipalities. Both groups recognized the importance of preventing taeniasis by avoiding the consumption of raw pork. However, specific knowledge related to the transmission and cause of cysticercosis was lacking among individuals in both groups.

Furthermore, higher knowledge levels were correlated with higher monthly family income. Approximately 54.2% of participants had an average household monthly income of 10,000 THB and above. Among these participants, most resided in the town municipality versus subdistrict municipalities. This suggests that participants living in town municipalities with higher socioeconomic status may possess higher knowledge levels regarding these parasitic diseases compared with those in subdistrict municipalities. However, this study showed no association between knowledge and residents’ characteristics. This finding is in contrast with those regarding other infectious diseases, such as a report by Zhang et al. (2018) that showed that participants in urban areas have significantly higher levels of knowledge regarding all aspects of COVID-19 (transmission, prevention measures, symptoms of infection, treatment, and prognosis; p<0.01), compared with their rural counterparts Therefore, efforts to raise awareness should target the general community in subdistrict municipalities as well as geographic disparities in knowledge levels. A similar study conducted in Uganda found that male farmers had greater knowledge about the disease than their female counterparts, possibly owing to men’s greater involvement in social gatherings and women’s greater involvement in domestic work. Additionally, farmers in more urbanized areas had more correct knowledge than those in rural areas [30]. Conversely, a study by Shongwe et al. (2020) in South Africa found no significant association between level of education, source of income, purpose of pig farming, and knowledge about neurocysticercosis. Similarly, there was no significant association between residence in a town, education level, income source, pig farming purpose, years engaged in pig farming, number of pigs kept by the farmer, and knowledge about porcine cysticercosis. However, those authors identified factors previously associated with the epidemiology of taeniasis and neurocysticercosis, including free-roaming pigs, lack of meat inspection, lack of knowledge about these diseases, and sewage spillage. Thus, education and training of farmers regarding the epidemiology of porcine and human cysticercosis are necessary to mitigate the risk of *Taenia* spp. infection among farmers and consumers [20].

In terms of practices, a significant portion of the population in Pak Chong district (89.7%) engaged in behaviors that are associated with taeniasis and cysticercosis. However, there were notable differences between individuals residing in the town municipality and those in subdistrict municipalities. Specifically, individuals living in subdistrict municipalities had a higher prevalence of risky practices related to cysticercosis compared with those in the town municipality [39]. This potentially resulted in diminished overall health outcomes. These practices included washing vegetables/fruits with water from natural sources such as rainwater, groundwater, and untreated rivers (5.3% and 1.9%, respectively). This could be attributed to easier access to natural water sources, particularly in isolated areas in subdistrict municipalities where tap or clean water may not be readily available. Contamination of the environment with *T*. *solium* eggs increases the likelihood of pigs and humans ingesting them when open water sources such as rivers, streams, wells, and lakes are used in households without proper boiling or decontamination measures [18]. Similarly, a study in rural communities of southern Thailand found that intestinal parasitic infections were closely associated with unsafe drinking water, poor sanitation, poor personal hygiene, poverty, and climatic conditions [40]. In contrast, Jorga et al. (2022) found low practice scores among participants and observed several poor practices, such as raw meat consumption, backyard slaughter, and open defecation, which can contribute to the spread of disease [22]. Additionally, our results showed that 31.9% of people (43.6% in the town municipality and 27.8% in subdistrict municipalities) consumed pig meat, and 1.7% had consumed meat with cysts, indicating that participants engaged in better practices regarding taeniasis. In comparison, a study in Burkina Faso found that 80.6% of people consumed pig meat, and 30.9% had consumed meat with cysts, which contributes to the spread of these diseases [38]. Raising awareness among the population about risks associated with the consumption of infected or undercooked pig meat could help reduce the spread of parasitic diseases.

Furthermore, only a small percentage (5.6%) of our study population reported routine deworming with anthelmintic medication on an annual basis, which can help limit transmission through pigs and the environment. Future studies should address the importance and benefits of anthelmintic drugs for both humans and pigs. Additionally, individuals in the town municipality exhibited better practices in terms of the prevention of taeniasis and cysticercosis. These participants were more likely to wash vegetables and fruits with tap water instead of natural water and to consistently wash their hands with clean water and soap after using the restroom or before handling food. Most individuals in the town municipality also had better practices regarding checking the appearance of pork meat before preparing it, although the difference was not statistically significant in comparison with residents of subdistrict municipalities. The practice score was negatively associated with age more than 60 years and living in a subdistrict municipality. Similarly, a study conducted among Iranian inpatients with type 2 diabetes found a negative correlation between age and practices, although older patients demonstrated a good level of knowledge and attitudes [41]. Furthermore, a study investigating KAP regarding COVID-19 among people in rural and urban areas of Cameroon revealed that rural residents exhibited poorer practices related to COVID-19 compared with urban dwellers. Specifically, rural inhabitants were less likely to avoid crowded places, wear face masks outdoors, and practice hand hygiene [42]. Our findings highlight a significant disparity in taeniasis and cysticercosis-related practices among individuals over 60 years old residing in subdistrict and town municipalities of Pak Chong. This difference could be attributed to variations in education level or a lack of knowledge about these diseases. It is also possible that their familiarity with certain eating habits leads them to overlook the adverse effects of consuming raw food. Consequently, future education programs should prioritize addressing practices among older participants residing in subdistrict municipalities to bridge this gap.

When comparing overall KP between the general population in the town municipality (n = 94) and subdistrict municipalities (n = 266), the findings revealed that the population in the town municipality had higher levels of accurate knowledge and better practices in comparison to the population in subdistrict municipalities. However, there was no significant association with KP, indicating similar knowledge levels among those with both good and poor practices. This might be because the study area is endemic for other parasitic infectious diseases, such as *O*. *viverrini* [43]. Therefore, the population in this area may be attempting to prevent these infections by avoiding the consumption of raw foods and performing other practices that are similar to those necessary to prevent taeniasis and cysticercosis. However, the survey revealed that 98.3% of participants were classified as having less accurate knowledge regarding these diseases. This reveals an important issue for health education in this area.

The implementation of health programs plays a pivotal role in modifying local cultural and habitual practices, leading to sustainable improvements in the environment of villagers. A compelling example of the effectiveness of this approach can be observed in work conducted by Alexander et al. (2012) in South India. Their study demonstrated that initial knowledge gaps and inadequate hygiene and sanitation practices were prevalent among the villagers. However, through health education interventions delivered in villages and schools that focused on the lifecycle of the pork tapeworm, the spread of taeniasis and cysticercosis, and prevention measures, significant improvements were achieved [28]. Similarly, Wandra et al. (2015) conducted a study in Indonesia, implementing a comprehensive approach to the prevention and control of taeniasis. Their research underscored the importance of combining a treatment program for human taeniasis carriers with pig vaccination as a crucial measure for successful control of taeniasis and neurocysticercosis in endemic areas [44].

These findings highlight the need to adopt a multi-faceted strategy that targets both human and animal hosts to effectively control and prevent these diseases. The results indicate that awareness campaigns and education programs can be instrumental in improving knowledge levels among the population. In the case of Pak Chong, our results indicate that efforts should be focused on improving knowledge about taeniasis and cysticercosis, particularly among elderly individuals in subdistrict municipalities, to enhance practices for infection prevention and control. Moreover, there should be an emphasis on health education among VHVs in Thailand, with a focus on personal hygiene and environmental sanitation. Developing awareness strategies and implementing training programs for parasite control and health promotion among community health workers are also crucial steps. Likewise, studies conducted in southern Thailand have suggested the importance of community awareness campaigns, appropriate training programs, and health promotion efforts to reach the most vulnerable populations and reduce the prevalence of parasite infections. Additionally, policymakers and public health officials should be encouraged to develop suitable control programs and strategies for VHVs, who play a key role in providing health education to the community [40, 45].

Our study has some limitations. The findings are based on cross-sectional data, limiting causal interpretations owing to the collection of influencing factors and outcomes at the same time. Further longitudinal studies should be conducted to evaluate KP and other factors related to taeniasis and cysticercosis. Furthermore, we focused on specific areas, limiting generalizability of the results to other regions. Therefore, further surveys should be conducted nationwide in both endemic and epidemic settings. Furthermore, the use of self-report interview methods may introduce response bias. To mitigate this bias, longitudinal interviews and qualitative studies should be implemented.

## Conclusions

In this cross-sectional study, we aimed to assess KP among the general population of Pak Chong District, Thailand regarding taeniasis and cysticercosis. The results indicated that most participants frequently engaged in appropriate practices related to prevention. This outcome might be associated with concern among the population about preventing infection with other parasitic diseases, despite lacking correct knowledge about taeniasis and cysticercosis. The present results showed that only a small percentage of participants had accurate knowledge about taeniasis and cysticercosis, and there was no significant association with KP concerning these parasitic infections. Furthermore, income levels and demographic factors, such as age and residing in a subdistrict municipality, had varying effects on KP. To address this gap in KP, public education interventions are recommended, particularly targeting individuals with lower socioeconomic status. These findings have implications for the development of targeted interventions and educational programs in Pak Chong District, particularly focusing on improving practices among the elderly population in subdistrict municipalities to effectively prevent and control these infections. Developing specific intervention strategies and assessing the long-term impact of health education programs on KP outcomes for taeniasis and cysticercosis control, as well as health promotion among the elderly population in subdistrict municipalities, would positively affect health outcomes.

## Supporting information

S1 Data (CSV)

## Abbreviations

KP: knowledge and practices
